# QTc is Prolonged in Patients with SSc and Associates with Skin Score

**DOI:** 10.31138/mjr.34.1.61

**Published:** 2023-03-31

**Authors:** Alireza Fatemi, Nafiseh Abdolahi, Mehrdad Aghaei, Hamidreza Azimi, Ahmad Mohammadi

**Affiliations:** 1 Student Research Committee, Golestan University of Medical Sciences, Gorgan, Iran,; 2 Golestan Rheumatology Research Center, Golestan University of Medical Sciences, Gorgan, Iran,; 3 Ischemic Disorders Research Center, Golestan University of Medical Sciences, Gorgan, Iran

**Keywords:** systemic sclerosis, prolonged QT, autoimmune disease, electrocardiogram, arrhythmia

## Abstract

**Background:**

Systemic sclerosis is an autoimmune disease characterised by endothelial dysfunction and fibrosis of the skin and internal organs. Cardiac involvement during systemic sclerosis can be primary or secondary to pulmonary arterial hypertension and renal pathology. Among the disorders in systemic sclerosis, prolongation of QTc time is also associated with more anti-RNA polymerase III antibodies, longer duration, and severity of disease.

**Methods:**

This case-control study was performed on 35 patients with systemic scleroderma who filled in the American Society of Rheumatism (ACR / EULAR criteria) and 35 healthy subjects prior to entering the study. Then, the QTc distance was extracted from the electrocardiogram and calculated using the formula. The measured QTc distance in the electrocardiogram, QTc> 440ms in men and QTc> 460ms in women, was defined as QTc long. The patients and the control group then underwent echocardiography, and changes in QTc interval and their relations with echocardiographic findings were evaluated.

**Results:**

The results of this study indicated a significant relationship between QTc distance in patients with scleroderma compared with healthy controls. There was also a significant relationship between QTc and Skin Score of patients. However, there was no significant correlation between QTc distance and age, duration of disease, Anti-Centromere, Anti-Scl70, and pulmonary artery pressure.

**Conclusion:**

This study concludes that patients with scleroderma are at high risk for cardiac conduction impairment. The only factor that significantly correlated with QTc was the Skin Score of the patients.

## INTRODUCTION

Cardiovascular diseases are one of the most important issues in Systemic sclerosis (SSc). The prevalence of SSc is 150–300 cases per million and is four times more common in women than in men.^1^ The occurrence of SSc and severe organ involvement, especially SSc-related cardiovascular disease, is associated with a poor prognosis and the possibility of death.^2^ The highly inconsistent presentation and prognosis of SSc makes it vital to identify patients at risk of death who may also have benefited from close monitoring and early treatment.^3^

About 95% of patients with SSc have been diagnosed with autoantibodies at the first diagnosis, and each of these autoantibodies are advantageous for diagnosing patients. Different autoantibodies have been correlated with different disease subtypes and with differences in disease severity, including the extent of internal organ manifestations, skin involvement, as well as determining the prognosis.^4^

The American College of Rheumatology (ACR) has established a set of diagnostic criteria for many autoimmune diseases that are used in clinics to interpret clinical and laboratory findings in patients with symptoms of connective tissue disease. Anti-Scl-70 and anti-centromere are two autoantibodies used by the American Society of Rheumatology to diagnose systemic scleroderma.

Cardiopulmonary manifestations in SSc patients encompasses dyspnoea, palpitations, chest pain, and heart failure, although most patients are asymptomatic at early stages and due to the lack of a specific diagnostic algorithm, the diagnosis is often postponement. Arrhythmia is one of the special events that also causes a poor prognosis.^5^ One-third of deaths in SSc patients occur due to the cardiac involvement, which is one of the leading causes, even though patients are often clinically silent.^6^ Previous studies remarkably reported that sudden cardiac death was the terminal event in 5% of SSc patients and was associated with skeletal myositis and ventricular arrhythmias in two large post-mortem analysis. Therefore, its prevention is very crucial in the management of these patients. One of the independent predictors of fatality presented in 25–75% of SSc patients is abnormal standard 12 lead electrocardiogram (ECG) which also requires special consideration.^5^

Therefore, early detection of heart abnormalities is very important. Several techniques for diagnosing asymptomatic cardiac abnormalities are available including magnetic resonance imaging, single-photon emission computed tomography, and cardiac computed tomography. These sensitive techniques can be used for both structural and functional scleroderma-related cardiac pathologies, but in addition to financial aspects, they require skills and learning that are not publicly available.^7^ It is therefore recommended that all SSc patients undergo echocardiography, which is a cheaper, more accessible, and non-invasive method. Echocardiography is useful to show changes that occur in the context of increased pulmonary artery pressure (PAP). It can also partially show myocardial abnormalities in the field of fibrosis and decrease myocardial perfusion or even pericardial effusion.^8^

The most frequent ECG abnormalities in SSc patients are represented by P wave abnormalities, non-specific abnormalities of ST-segment and T wave, QRS micro voltage, and more subtle changes like the low values of the heart rate variability parameters, QT dispersion (QTd) increase.^9^ The prolongation of QTc interval has been also observed in patients with SSc without cardiac manifestations and in the absence of echocardiographic abnormalities which can lead to life threatening tachyarrhythmias.^10^

In fact, in patients with SSc, an association was observed between QTc prolongation and capillaroscopic pattern with finger ulcers and skin thickness score.^8^ Anti-RNA polymerase III antibodies and longer duration of disease are also related to the prolongation of QTc interval among disorders in SSc patients.^8,11^

Considering all the above conditions and the importance of QTc interval and its relationship with systemic conflicts, therefore, this study was designed to determine this interval and its relationship with disease-related factors.

## METHOD

### Study subjects

According to the literature review, the mean (standard deviation) QTc interval in patients with scleroderma was 422.4 (47.1).^12^ Considering the default difference of at least 10% between the QTc interval in patients with SSc with the control group^8^ and the confidence interval level of 0.95 and 90% power, using the following formula.


$$n=\frac{2\delta^{2}{\left({\text{Z}}_{1-\frac{\alpha }{2}}+{\text{Z}}_{1-\beta }\right)}^{2}}{{\left(\mu_{1}-\mu_{2}\right)}^{2}}$$

The calculated sample size in each group is 27 people. Finally, considering about 20% of the loss in the samples, the final sample volume in each group was determined to be 35 individuals.

This case-control study was performed on 35 patients with SSc referred to the private and public rheumatology clinics in Golestan province who met the criteria for diagnosis and classification of the American Rheumatism Association (The ACR/EULAR criteria for the classification of SSc)^13^ and 35 healthy individuals before admission. After obtaining informed consent from the subjects and assuring theme, electrocardiogram was taken from each of the patients and individuals from the control group. Scleroderma patients with known coronary artery disease (CAD) through coronary artery bypass graft (CABG), diabetes, kidney failure, liver or thyroid disorder, anaemia, and patients receiving beta-blocker, antiarrhythmic drugs were excluded from the study.

The control group consisted of healthy individuals who matched patients in terms of age and gender. Also, at the time of admission, they didn’t have any of the CAD known through CABG, diabetes, kidney failure, liver or thyroid disorder, anaemia, and patients without beta-blocker and antiarrhythmic drugs.

### Statistical assessment

The patients were examined, and their skin scores were determined. Echocardiography was also recorded from both case and control group. Patients’ blood samples were also taken, and antibodies were sent for laboratory evaluations.

### Statistical analysis

After assembling the study participants’ information, the data were entered in SPSS software version 16 and the information was summarised and reported in tables and statistical charts. To investigate the relationship between FC level and the studied independent variables; In case of statistical assumptions of normality and homogeneity of variance, independent t-test and analysis of variance were used, otherwise equivalent nonparametric tests such as Mann-Whitney and Kruskal-Wallis were applicated.

### Ethical approval

This work was carried out under research program of Golestan University of Medical Sciences with the ethical code ir.goums.rec1396.262.

## RESULTS

A total of 70 people were included in the study. The subjects were divided into two groups in terms of age: under 40 years old as young group and over 40 years old as elderly group. the mean age of the subjects was 44.34 and their standard deviation was 12 years. 2 (2.9%) were male and 68 (97.1%) were female of the total subjects.

All participants were studied in two groups of patients and control group. 35 (50%) in the patient group and 35 (50%) in the control group entered the study of the total subjects. In the group of patients, 1 (2.9%) of the subjects were male and 34 (97.1%) were female as same as the control group. Also, the number of people in both young and old groups was 16 in the patient group and 19 in the control group.

The amount of QTc in the subjects varied between 380 ms to 593 ms. The mean QTc in the subjects was 446.71 ms and the standard deviation was 54.17 ms.

According to the findings of **Table 1**, the amount of QTc in patients is significantly higher than the control group.

**Table 1.** Mean QTc in the studied groups. Nonparametric used test: independent T test.

**Studied groups**	**Mean QTc**	**Standard deviation**	**P value**
Total subjects	424.21	36.85	0.001
Patients	438.71	51.33	
Control group	409.71	22.37	

The amount of mean QTc were also measured in two age groups of young subjects and elderly subjects.

The mean QTc and standard deviation were 440.56 and 50.33 in the young subjects, respectively, while in the elderly group these values were 451.89 and 57.35, respectively. According to the findings of **Table 2**, no significant relationship was observed between QTc and different age groups (P=0.39).

**Table 2.** Mean QTc in the age groups. Nonparametric used test: independent T test.

**Age groups**	**Mean QTc**	**Standard deviation**	**P value**
Young	440.56	50.33	0.39
Elderly	451.89	57.35	

Besides, we divided 35 patients with scleroderma into Prolong QT and No Prolong QT groups and compared the mean and standard deviation of each of the variables in these two groups.

According to the findings of **Table 4**, no significant relationship was observed between QTc and different age groups, disease duration (Pearson correlation coefficient −0.12 and P = 0.48), anti-centromere (Pearson correlation coefficient 0.13 and P = 0.47), Anti-Scl70. (Pearson correlation coefficient 0.02 and P = 0.93), and PAP (Pearson correlation coefficient 0.04 and P = 0.82), But there was a remarkable correlation between QTc and skin score (Pearson correlation coefficient 0.603 and P = 0.006). Also, **Table 3** shows all basic epidemiologic and clinical data of SSc patients without dividing subjects to prolonged and non-prolonged QTc.

**Table 3.** The demographic and clinical parameters of the patients and healthy control group of the study.

**Variables**	**Mean**	**Standard deviation**
Age^*^	44.51	12.15
Disease Duration^**^	9.95	7.65
Skin score^**^	12.05	8.4
Anti-Scl70^****^	151.99	178.25
Anti Centromere^**^	41.45	69.15
PAP^*^	28.55	8.65

**Table 4.** Demographic and clinical parameters of the patients and healthy control group of the study divided to prolonged and non-prolonged QT groups.

**Variables**	**Prolonged QT**		**Non-Prolonged QT**		**P value**
	**Mean**	**Standard deviation**	**Mean**	**Standard deviation**	
Age^*^	43.91	11.7	45.12	12.6	0.69
Disease Duration^**^	11.5	8.5	8.4	6.8	0.29
Skin score^**^	9.8	8.9	14.3	7.9	0.21
Anti Scl70^****^	137.88	162.3	166.1	194.2	0.68
Anti Centromere^**^	48.4	71.8	34.5	66.5	0.58
PAP^*^	28.9	7.5	28.2	9.8	0.85

Nonparametric used test:

* Independent T-test,

** Mann–Whitney test

## DISCUSSION

QTc prolongation is considered as a significant predictor for cardiovascular events associated with potentially lethal complications. Therefore, many studies were conducted to evaluate QTc prolongation in systemic sclerosis patients. In a similar study conducted by Gigante et al.^14^ in 2015, the mean QTc interval was significantly (P <0.001) higher in patients with SSc compared to the healthy control group.

Also, in the study of Roseta et al.,^8^ the mean QTc interval was significantly higher (P <0.0001) in patients with scleroderma than the healthy control group (447 vs. 386). The study by Massie et al.^12^ also confirms the findings of the above studies. (Mean QT interval in SSc patients was 422.4 ms (.1 47.1) and 25% of prolonged patients had QT). A study by Orcun Ciftc et al.^15^ Also showed that patients with diffuse scleroderma may be asymptomatic but have cardiac repolarisation abnormalities and autonomic disorders.

This study and all the mentioned studies showed that prolonged QT is very common in SSc patients. It can be assumed that at least part of the reason for the high incidence of prolonged QT in SSc patients is extensive cardiac fibrosis. Disorders increase the period of ventricular repolarization. However, even if the effect of fibrosis on the intraventricular conduction system is confirmed, its effect on repolarization properties is unknown. Recent evidence, however, suggests that transforming growth factor (TGF)-beta, a key factor in the pathogenesis of SSc, may cause electrical changes in the heart, due to its profoundly influence by the transcription and function of specific ion channels expressed by keratinocytes.

Mean QTc in men (two individuals), was 408 and the standard deviation was 28.28. The mean QTc in women (n = 68) was 447.85 and the standard deviation was 54.44. The test results showed that QTc values were not significantly different between men and women. (P = 0.32). No significant relationship was observed between QTc and different age groups. Due to the small statistical population of men in this study and in similar studies, the above statistical findings about the correlation or lack of correlation between gender and Prolong QT cannot be reliable.

In this study, no significant correlation was observed between QTc and disease duration. (Pearson correlation coefficient −0.12 and P = 0.48). In the study of Roseta et al.^8^, No significant relationship was found between QTc distance and disease subspecies, disease duration, disease activity index and disease severity criteria. In the study by Massie et al.,^12^ There was a direct relationship between Prolong QT with longer duration of illness and higher severity of the disease. However, the relationship between Prolong QT and disease duration was inverse in the study of Foocharoen et al.^16^ In this study, there was a significant correlation between QTc and skin score. (Pearson correlation coefficient 0.603 and P = 0.006) This finding is consistent with the findings in the Roseta et al.^8^ study that there is a positive correlation (P <0.05) between QTc and mRSS. It was reported that Rodnan’s higher skin score could be a predictor of heart attack in SSc patients.

No significant correlation was observed between QTc with Anti-centromere and Anti-Scl-70. (Pearson correlation coefficient 0.13 and P = 47.0), (Pearson correlation coefficient 0.02 and P = 0.93). The study by Massie et al.^12^ Also showed that Anti RO, both separately and simultaneously, did not interact with Prolong QT. However, there was a significant correlation between the presence of anti-RNA polymerase III and Prolong QT antibodies. However, a study by Nakamura et al.^17^ Suggests that increasing the QTc interval, which ultimately increases the duration of cardiac repolarization, may be due to the inhibitory effect of anti-Ro antibodies on KCNH2 channels (HERG), which plays an important role in cardiac repolarisation by IKR.

However, no significant correlation was observed between QTc and PAP, but the average PAP in both prolonged QT and No Prolong QT patients is about 28mhg, which is higher than normal (Pearson correlation coefficient 0.04 and P = 0.82).

## CONCLUSION

The results of this study indicate a significant relationship between QTc interval in patients with scleroderma compared with the healthy control group. There was also a remarkable relationship between QTc and skin score of patients While no significant relationship was observed between QTc interval and age, sex, disease duration, Anti-Centromere, Anti-Scl-70, and PAP.

Determining the lack of a definite relationship between gender and QTc interval is difficult due to the very low number of male patients with scleroderma in this study and generally the low incidence of scleroderma in men. In general, it can be concluded from this study that patients with scleroderma, despite being asymptomatic, are at high risk for cardiac conduction disorders, which is not related to age, duration of disease, and laboratory values. The only factor that was significantly associated with QTc interval in this study was patients’ skin score.

In conclusion, patients with scleroderma with higher skin score are in greater need for early screening for conduction disorders of the heart.
